# Ignoring Non-ignorable Missingness

**DOI:** 10.1007/s11336-022-09895-1

**Published:** 2022-12-20

**Authors:** Sophia Rabe-Hesketh, Anders Skrondal

**Affiliations:** 1 Norwegian Institute of Public Health, 2121 Berkeley Way, Berkeley, CA 94720 USA; 2 University of Oslo, Oslo, Norway; 3 University of California, Berkeley, 2121 Berkeley Way, Berkeley, CA 94720 USA

**Keywords:** data deletion, MAR, make-MAR, missing data, *m*-graph, ordered factorization, protective estimation

## Abstract

The classical missing at random (MAR) assumption, as defined by Rubin (*Biometrika* 63:581–592, 1976), is often not required for valid inference ignoring the missingness process. Neither are other assumptions sometimes believed to be necessary that result from misunderstandings of MAR. We discuss three strategies that allow us to use standard estimators (i.e., ignore missingness) in cases where missingness is usually considered to be non-ignorable: (1) conditioning on variables, (2) discarding more data, and (3) being protective of parameters.

Missing data are pervasive in empirical research, and a wide range of methods have been developed to address this problem. We recommend Little and Rubin (2020) and Allison (2002) for gentle introductions to these methods and Molenberghs and Kenward (2007) and Daniels and Hogan (2008) for more technical treatments.

The aim of this IMPS 2015 presidential address is to discuss under what assumptions and with what modifications to data or models, standard estimators, such as maximum likelihood, are valid, so that the missingness process can be ignored. The term “ignorable” was used in the seminal Rubin (1976) article to describe assumptions regarding the missingness process (missing at random (MAR) and distinctness) that are needed to obtain valid inferences, ignoring the missingness process. If missingness is ignorable, we can estimate the model of interest, ignoring the missingness process, by defining the likelihood for all available (AA) data. This AA-data likelihood can be used for maximum likelihood or Bayesian estimation, and the latter can be implemented via multiple imputation (without an auxiliary model). Following Little and Zhang (2011), we will use “ignorable likelihood” (IL) as the umbrella term for these three approaches.

The term “ignoring” in the first part of this paper’s title means not modeling the missingness mechanism, whereas the term “non-ignorable” in the second part of the title means that Rubin’s assumptions for ignorability are violated. Instead of modeling the “non-ignorable” missingness mechanism, some modifications to data or models may be necessary before proceeding with standard IL methods. These modifications include conditioning on variables, discarding more data, switching from random-effects to fixed-effects estimation, or introducing additional parameters in the model to “protect” the parameters of interest. Which approach to take does not depend on any parametric assumptions regarding the missingness process but is determined by conditional independence assumptions between missingness (or selection) indicators and the variables of interest.

Our plan is as follows. In Sect. 1 we introduce modifications to Rubin’s MAR assumption and define R-MAR, an assumption that allows valid frequentist inference by IL approaches. In Sect. 2 we briefly describe three strategies for valid inference when R-MAR is violated, and these strategies become the topics of Sects. 3, 4, and  5. Whereas C-MAR, the modified MAR requirement when conditioning on variables (Strategy 1, Sect. 3), has been discussed in the literature, confusion about this requirement persists. The idea of discarding more data to relax R-MAR (Strategy 2, Sect. 4) is new, to our knowledge. We show how it relates to sequential estimation based on Mohan, Pearl, and Tian’s (2013) ordered factorization theorem and that it is preferable to sequential estimation when IL methods are applicable. Section 4 gives an overview of protective estimation (Strategy 3, Sect. 5) where estimators and models are selected to protect specific parameters from being inconsistently estimated due to missing data. In Sect. 5 we end with some concluding remarks.

## MAR, its Modifications, and Ignorability

### MAR and its Modifications

We assume throughout that we have a parametric model for our variables of interest with parameters $\varvec{\theta }$ and that we would like to make inferences regarding (possibly a subset) of these parameters. We also assume that the model is correctly specified so that we can focus on the impact of missing data. Let ${{\textbf {U}}}_i$ be the vector of all variables in the model for unit *i*, e.g., ${{\textbf {U}}}_i=(X_i,Z_i,Y_i)^{\prime }$, with realized values ${{\textbf {u}}}_i=(x_i,z_i,y_i)^{\prime }$. A separate missingness or selection process determines which of the variables are observed for which units. The vector of selection indicators ${{\textbf {S}}}_i$ has elements equal to 1 if the corresponding variable is observed for unit *i* and 0 if it is missing, e.g., ${{\textbf {S}}}_i=(S_i^x,S_i^z,S_i^y)^{\prime }$ with 4 realized values ${{\textbf {s}}}_i=(s_i^x,s_i^z,s_i^y)^{\prime }$. We will occasionally say that a variable is “selected” for a unit, meaning that it is not missing.

Using the notation ${{\textbf {u}}}_i^{\text {obs}}$ for the sub-vector of ${{\textbf {u}}}_i$ containing the variables that are observed, i.e., the variables for which the corresponding elements in ${{\textbf {s}}}_i$ are 1, Rubin’s (1976) MAR assumption can be written as

$$\begin{aligned} P({{\textbf {s}}}_i|{{\textbf {u}}}_i)=P({{\textbf {s}}}_i|{{\textbf {u}}}_i^{\text {obs}}). \end{aligned}$$

In words, the probability that the missing variables are missing, given the realized values of all observed variables, is unchanged, regardless of what values are substituted for the missing variables (Rubin, 1976; Seaman et al., 2013).^1^ Rubin (1976) calls the missingness ignorable if MAR holds and if the parameters of the missingness process are distinct from the parameters of the model of interest. Under these assumptions, direct maximum likelihood inference (without frequentist claims) and Bayesian inference are valid. Rubin (1976) did not define missing completely at random (MCAR), but it later became understood (e.g., Mealli & Rubin, 2015) to mean $P({{\textbf {s}}}_i|{{\textbf {u}}}_i)=P({{\textbf {s}}}_i)$.

An important paper by Seaman et al. (2013) points out that Rubin’s MAR definition has been widely misunderstood by not recognizing that it refers to the realized selection indicators and the realized data. Instead, MAR has been interpreted as a conditional independence statement for random variables, , where ${{\textbf {U}}}_i^{\text {mis}}$ is the sub-vector of ${{\textbf {U}}}_i$ containing the variables that are not observed and ${{\textbf {U}}}_i^{\text {obs}}$ is the corresponding sub-vector of variables that are observed. Definitions of MAR based on random variables rather than their realized values can be interpreted as a stricter requirement than Rubin’s MAR, namely that Rubin’s MAR should hold in *repeated samples*. Seaman et al. (2013) show that *frequentist* likelihood inference ignoring the missingness process requires that MAR *always* holds (in repeated samples) and calls that assumption “everywhere MAR.” Mealli and Rubin (2015) adopt the same definition and suggest the term “always” instead of “everywhere.” We will use the acronym A-MAR for *always* MAR.

A problem with these MAR assumptions is that different units have different variables in ${{\textbf {U}}}_i^{\text {obs}}$ (see also Schafer & Graham, 2002), and it rarely makes sense to assume that a variable $X_i$ affects selection of other variables only if it is observed, $S_i^x=1$. An exception would be if *X* only affects selection when it has been realized or revealed to the individual (e.g., failing an educational assessment). Generally, a more plausible MAR condition therefore is what we call *realistic* MAR (R-MAR), where missingness cannot depend on any variable that *can* be missing. Pothoff et al. (2006) call this assumption MAR+, Greenland and Finkle (1995) refer to it as “stratified MCAR,” and Mohan et al. (2013) define their MAR assumption this way. If both *X* and *Y* can be missing and *Z* is always observed, the assumption becomes1Note that it is now valid to write the assumption as a conditional independence statement. In contrast, the conditional independence statement  is problematic, as pointed out by Seaman et al. (2013), because ${{\textbf {U}}}_i^{\text {mis}}$ is a function of ${{\textbf {S}}}_i$ (in the sense that ${{\textbf {S}}}_i$ determines which elements of ${{\textbf {U}}}_i$ are missing) and can therefore not be conditionally independent of it.

In their MAR definition, Mohan et al. (2013) refer to variables like *X* and *Y* that have missing values as “partially observed” and to variables like *Z* that have no missing values as “fully observed” without discussing what would happen in repeated samples. In contrast, Mealli and Rubin (2015) use the term “always observed” to clarify that this is not just what happened in the realized data but that it is an assumption regarding the missingness mechanism. They prove that when the units are exchangeable, A-MAR implies what we call R-MAR. However, as Mealli and Rubin (2016) point out in an erratum, this is true only if the selection indicators ${{\textbf {S}}}_i$ are mutually independent given ${{\textbf {U}}}_i$, which is not required by A-MAR or R-MAR. For instance, A-MAR with exchangeable units allows for the possibility that selection $S_i^x$ of $X_i$ depends on $Y_i$ only when $Y_i$ is observed, $S_i^y=1$. These kinds of processes seem odd, which is the reason for our term *realistic* MAR, but we will make use of such a process in Sect. 4 to justify one of our approaches, namely discarding more data.

### Ignorability and IL Methods

A missingness process is ignorable if it is valid to base inferences on the AA-data likelihood, ignoring the missingness process, instead of the joint likelihood of the data and the missingness process. The assumptions required for ignorability depend on the kind of inference we wish to make, such as direct likelihood, Bayesian, or frequentist likelihood inference. Seaman et al. (2013) show that A-MAR, together with distinctness of the parameters of the missingness process and the model of interest, is ignorable for frequentist likelihood inference. The reason is that the likelihood of the data is proportional to the joint likelihood of the missingness process and the data, and this is true not just for the realized data but also in repeated samples. Hence, the point estimates and *observed* information matrix based on the likelihood of the data (ignoring the missingness process) are identical to those based on the joint likelihood of the data and missingness process *in each repeated sample*. Therefore tests and confidence intervals will have the same frequentist properties for both approaches. Using analogous arguments, Bayesian point estimators and credible intervals have the same repeated sampling properties whether they are based on the likelihood of the data or the joint likelihood of the data and missingness process. Because R-MAR implies (and is stricter than) A-MAR, R-MAR is also ignorable in the same sense.

The likelihood of the data (ignoring the missingness process) is the joint likelihood of the variables, integrated over the missing data. For simplicity, consider three variables, *X*, *Z*, and *Y*, where *Z* is always observed. The log-likelihood contribution of a unit can then be written as (suppressing the *i* subscript)

2 $$\begin{aligned} L^{\text {joint}}\ =\ {}&s^xs^y\text {ln}P(x,z,y) \nonumber \\&+ s^x(1-s^y)\text {ln}P(x,z) + (1-s^x)s^y\text {ln}P(z,y) \nonumber \\&+ (1-s^x)(1-s^y)\text {ln}P(z). \end{aligned}$$

Each term corresponds to a missingness pattern, with $(s^x,s^y)$ equal to (1,1) for the first term, (1,0) for the second, (0,1) for the third, and (0,0) for the final term. Correspondingly, *P*(*X*, *Z*, *Y*) is the joint distribution of all variables, *P*(*X*, *Z*) is the marginal joint distribution of *X* and *Z*, integrating out *Y* because it is missing, and similarly for the remaining terms. For each pattern, we make use of all available data, as described for a multivariate normal distribution by Anderson (1957). As mentioned in the introduction, when this likelihood is used in maximum likelihood or Bayesian estimation (including multiple imputation without an auxiliary model), we will use the umbrella term “ignorable likelihood” (IL) method.

**Fig. 1:**
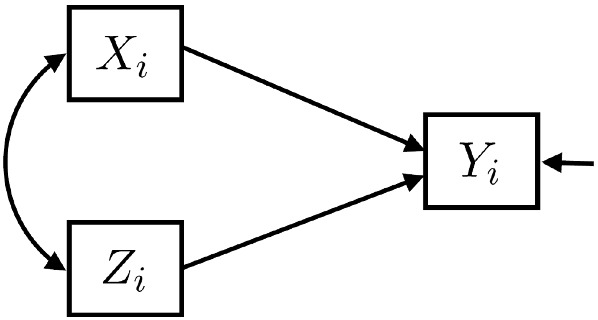
Linear regression model via multivariate model, SEM.

Importantly, R-MAR treats all variables in ${{\textbf {U}}}$ as response variables, with the implicit assumption that the likelihood is defined as in (2). However, typically the model of interest is a regression model (in a general sense, e.g., linear, logistic, multilevel, quantile, etc.) for *Y* given *X* and *Z*. To make use of the R-MAR assumption, we can embed this model within a multivariate model for ${{\textbf {U}}}$. In the case of linear regression, this is easy to do by specifying a linear structural equation model (SEM) as shown in Fig. 1 where the parameters of interest are the coefficients for the paths $X\rightarrow Y$ and $Z\rightarrow Y$. Maximum likelihood estimation for linear SEMs based on AA data was discussed in detail by Muthén et al. (1987) and Allison (1987) for the case of few missingness patterns (multiple group approach) and Arbuckle (1996) for the general case. If all variables are categorical, loglinear models can be used in an analogous way. Under R-MAR, all IL methods will have the same frequentist properties as the corresponding approaches based on the joint likelihood of the data and selection indicators.

In addition to the various definitions of MAR discussed in Sect. 1.1 that have been a source for confusion, there are three other sources of confusion, each of which suggests a strategy or guiding principle that allows us to ignore missingness processes that are usually understood to be non-ignorable. Section 2 gives an overview of these strategies, and the following sections provide more details on each strategy.

## Three Strategies

### Strategy 1: Condition on (Functions of) Variables

An important source of confusion is that the MAR assumptions (Rubin’s MAR, A-MAR, or R-MAR) are relevant only for inferences regarding the *joint* distribution of ${{\textbf {U}}}$. However, by far the most common types of analyses are regression models for one response variable given a set of covariates. Conditioning on covariates automatically results in units with incomplete data to be discarded, sometimes called listwise deletion or complete-case analysis. Contrary to common belief, such an approach does not require any of the MAR assumptions but rather an assumption that we will call *conditional* MAR, or C-MAR, that is more lenient than R-MAR. Unfortunately, it is common practice to apply a univariate MAR condition to each variable, such as incorrectly requiring that missingness of a covariate *X* cannot depend on *X* itself, given the other variables. However, this misconception can lead to adoption of approaches that fail when *X* directly affects its own missingness.

In latent variable models, conditioning on sufficient statistics for the latent variables (conditional maximum likelihood estimation) means that C-MAR can be relaxed further to allow selection to depend directly on the latent variables.

### Strategy 2: Discard More Data

MAR allows selection of one variable to depend on selection of another variable for the same unit. This is again due to the multivariate definition of MAR where ${{\textbf {S}}}_i$ is a vector of all selection indicators for unit *i*, so these indicators can be dependent. This issue is rarely discussed and, in fact, Mealli and Rubin (2015) neglected this possibility in their theorem. It turns out that we can interfere with the missingness process, by discarding data in some variables for those units for which other variables are missing, making the selection indicators more dependent, and thereby *making* the process MAR. We refer to this approach as M-MAR (for *make* MAR). By imagining that we would discard data in this way in repeated samples, so that it becomes part of the missingness process, the process becomes A-MAR and frequentist likelihood inference becomes valid. We can alternatively think of the data deletion as being part of the estimator. We show that there is a close connection between our M-MAR approach and Mohan et al.’s (2013) ordered (or sequential) factorization theorem.

### Strategy 3: Be Protective of (Subsets of) Parameters

Violation of MAR conditions (e.g., A-MAR, R-MAR, C-MAR), i.e., the problem of missing not at random (MNAR), does not imply that *all* parameters are estimated inconsistently when ignoring the missingness mechanism. Some estimators may be consistent for the parameters of interest. A well-known example is binary logistic regression for case–control data, where cases (with response variable equal to 1) and controls (with response variable equal to 0) have different probabilities of inclusion in the sample, which violates C-MAR. Nevertheless, standard maximum likelihood estimators of the regression coefficients and corresponding odds ratios are consistent, although the estimator of the intercept is not.

We can sometimes modify our model or estimation method to protect the parameters of interest from being estimated inconsistently, a strategy we call protective estimation (Skrondal & Rabe-Hesketh, 2014). For example, in binary longitudinal data, different kinds of conditional maximum likelihood estimators can be used to protect the odds ratios of interest. These results also take advantage of conditioning (Strategy 1) and can involve discarding some data (Strategy 2).

The next three sections discuss each of the strategies in more detail.

## Strategy 1: Condition on (Functions of) Variables

### Complete-Case (CC) Regression Analysis

If we are only interested in the conditional distribution *P*(*Y*|*X*, *Z*), as in a regression model, it seems cumbersome to specify and estimate a multivariate model for *X*, *Z*, and *Y* and use the joint likelihood in (2) for IL estimation as described in Sect. 1.2. Instead, we may want to use the likelihood conditional on the covariates. The only units that make contributions to the likelihood conditional on *X* and *Z* are those units that have complete data. Complete-case (CC) analysis refers to analyzing the subsample of individuals with complete data, sometimes called listwise deletion. If both *X* and *Y* can be missing, whereas *Z* is always observed, as in Sect. 1.2, the log-likelihood contribution from a unit becomes

$$\begin{aligned} L^{\text {cond}}\ =\ s^xs^y\text {ln}P(y|x,z). \end{aligned}$$

Due to the conditioning on covariates, MAR definitions are no longer useful, as also pointed out by White and Carlin (2010). In fact, we can *relax* the R-MAR assumption and define C-MAR aswhere $C=S^xS^y$ is an indicator for being in the CC sample. In a longitudinal setting, Little (1995) calls this assumption covariate-dependent missingness (or dropout).

This condition allows missingness of *X* to depend on *X* itself, given the other variables. For instance, if *X* is income, then whether income is reported can depend on income (and other covariates). Comparing C-MAR with R-MAR shows that there are situations where CC regression is valid and (multivariate) IL methods are not. Specifically, in any situation where missingness of *X* or *Y* depends on either *X* or *Y*, IL methods will not be valid, but CC regression will be valid as long as missingness of *X* or *Y* does not depend on *Y* (given *X*).

**Fig. 2:**
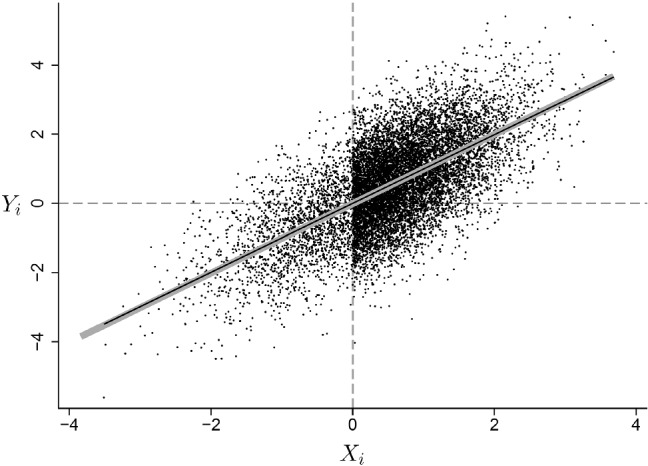
CC regression is consistent if , as long as .

Figure 2 illustrates the scenario where *X* is likely to be missing when it is less than zero and never missing when it is greater than zero (and here there is no other covariate *Z*). The ordinary least squares regression line (in black) coincides with the true regression line (thick gray line) because the distribution of *Y* given *X* is the same in the selected sample as in the full sample, $P(Y|X,S^x=1)=P(Y|X)$, and because the selected sample is so large that the least squares estimate is very precise. Selection just thins out the scatterplot to the left of zero but keeps the conditional distribution intact.

**Fig. 3:**
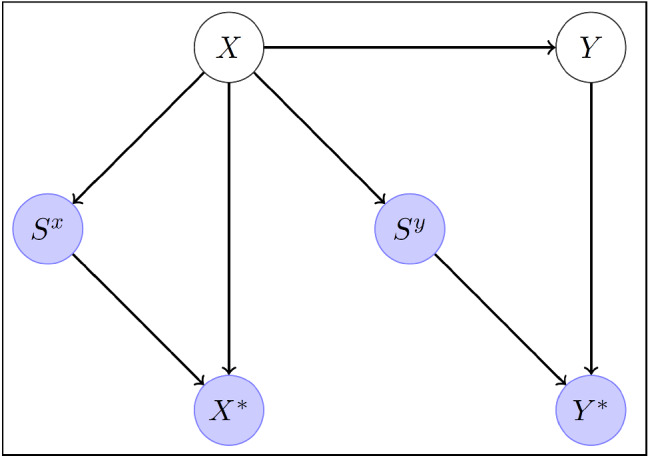
*m*-graph for C-MAR.

It is important to note that, while selection is associated with *Y* here, it is independent of *Y* *given X*, and therefore satisfies the C-MAR condition. Mohan et al. (2013) formalize this way of reasoning by representing the missingness process via directed acyclic graphs (DAGs) that they call Missingness Graphs or *m*-graphs. Conditional independence relations can then be derived by *d*-separation (e.g., Pearl, 2009). Figure 3 [same as Figure 1(c) in Mohan et al. (2013)] is an *m*-graph that satisfies C-MAR. There is no *Z* here and both *X* and *Y* are not always observed, as indicated by hollow circles. The variables $S^x$ and $S^y$ are caused by *X*, as shown by the paths from *X* to these variables, and they are fully observed (filled circles). The fully observed “proxy” variable $X^*$ equals *X* when the selection indicator $S^x=1$ and equals a symbol for missing, such as “NA” or “.”, otherwise. So $X^*$ is determined by the combination of *X* and $S^x$, as indicated by the two paths $X\rightarrow X^*$ and $S^x\rightarrow X^*$ and similarly for $Y^*$. The proxy variables and selection indicators are always observed and constitute the data. The question is whether we can estimate a given quantity or estimand (referred to as “query” by Mohan et al., 2013) from the data consistently. With the implicit assumption that all variables are categorical, Mohan et al. (2013) discuss estimation (or “recovery”) of the joint distribution *P*(*Y*, *X*) or conditional distribution *P*(*Y*|*X*) from the observed data. It follows from the graph that , so that $P(Y|X) = P(Y^*|X^*,S^x=1,S^y=1)$. Therefore, we can recover the conditional distribution from the observed data by estimating it in the CC sample. However, we cannot recover *P*(*X*) to obtain the joint distribution because of the path $X \rightarrow S^x$.

It has been pointed out frequently that CC regression is valid if missingness depends on the covariates, as long as it does not depend on the response variable given the covariates (e.g., Dardanoni et al., 2011; Jones, 1996; Little, 1992; Little & Rubin, 2020, p. 49; Seaman et al., 2013; Wooldridge, 2010, p. 796). Nevertheless, MCAR is often said to be necessary for valid CC regression (e.g., King et al., 2001; Molenberghs et al., 2004; Molenberghs & Kenward, 2007, p. 43). One reason for this confusion may be that covariates are sometimes not treated as random variables. For example, Diggle and Kenward (1994) define “completely random dropout” and “random dropout” in longitudinal data only in terms of whether dropout depends on current or previous values of the *outcome* variable (without conditioning on covariates). Another reason is that missingness that depends on covariates only is sometimes defined as MCAR (e.g., Daniels & Hogan, 2008, p. 92; Laird, 1988).

Believing that MCAR is necessary would erroneously lead to rejecting CC regression analysis based on the path from *X* to $S^y$ in Fig. 3 (even if there is no path from *X* to $S^x$). Then relying on the A-MAR assumption would lead to adoption of IL inference for the multivariate model. However, such an approach will likely be inconsistent because it is not realistic that *X* affects selection of *Y* only when *X* is observed. CC regression, in contrast, would yield valid inferences, even with the additional path from *X* to $S^x$. That multiple imputation can be invalid when CC regression is valid does not appear to be widely known although it has been pointed out repeatedly (e.g., Allison, 2000; Bartlett et al., 2014; Little & Zhang, 2011; White & Carlin, 2010).

Another common belief is that missingness of a covariate *X* in a regression model cannot depend on *X* itself given the other variables. This misconception appears to arise from falsely assuming that a univariate version of A-MAR must hold for each variable. Specifically, for each variable $V_i$, it is sometimes assumed to be necessary for valid inference that $P(S_i^v| V_i, {{\textbf {U}}}_i \backslash V_i) = P(S_i^v| ({{\textbf {U}}}_i \backslash V_i)^{\text {obs}})$, where $\backslash V_i$ means “excluding $V_i$.” This assumption is clearly violated in the scenarios depicted in Figs. 2 and 3 which satisfy C-MAR and hence produce valid inferences for regression models. Both Enders (2010, p. 11, 13) and Allison (2002, p. 4) define MAR in this univariate way and, when discussing that MAR is needed for ignorability, do not mention that this is so only for a multivariate model. Readers can find remarks elsewhere in these books that the univariate MAR assumption is not required for covariates in CC regression.

### Hybrid CC and AA Analysis: Subsample Ignorable Likelihood

As discussed in Sect. 3.1, CC regression is consistent if selection of any covariate in the model depends on the covariate itself, in contrast to inferences regarding the joint distribution of ${{\textbf {U}}}$ via IL methods. Little and Zhang (2011) therefore suggest a hybrid approach. Denoting the subset of covariates suspected of affecting their own selection as *W*, they assume that C-MAR holds for these variables, , where *Z* is completely observed variables (assumed to be always observed), and the variables in *X* are partially observed covariates, assumed not to affect their own selection. The subsample of units with complete data for *W* is then analyzed using IL methods based on the likelihood for *P*(*Y*, *X*|*Z*, *W*), under the assumption that MAR or A-MAR holds for selection of *X* and *Y*, given *W* and $S^W$. Little and Zhang (2011) write the assumption as $P(S^x,S^y|Z,W,X,Y,S^W)=P(S^x,S^y|Z,{{W}},X^{\text {obs}},{{Y^{\text {obs}}}},S^W)$. Note that this hybrid approach can also be viewed as an example of Strategy 2 to discard more data.

### Fixed Instead of Random Effects for Longitudinal or Clustered Data

We now consider longitudinal data where units $j=1,\ldots ,N$ are observed at $n_j$ occasions $i=1,\ldots ,n_j$. The variables $Y_{ij}$ and $X_{ij}$ are time-varying and $Z_j$ is time invariant. A linear random-intercept model can be written as

3 $$\begin{aligned} Y_{ij}\ =\ \alpha + \beta X_{ij} + \gamma Z_{j} + \zeta _j +\epsilon _{ij}, \end{aligned}$$

where $\zeta _j$ is a random intercept or latent variable and $\epsilon _{ij}$ an error term. Typically, it is assumed that $\zeta _j\sim N(0,\psi )$ and $\epsilon _{ij}\sim N(0,\theta )$. Associated with each variable is a selection indicator $S_{ij}^y$, $S_{ij}^x$, and $S_j^z$. The same model can also be used for cross-sectional clustered data, but we will use longitudinal-data terminology for concreteness.

Let $C_{ij}=S_{ij}^yS_{ij}^xS_j^z$ be the complete “case” indicator (where a “case” is a unit-occasion combination), taking the value 1 if all variables in the model are observed for unit *j* at occasion *i* and zero otherwise. We use vectors for the variables associated with a subject *j* across all $n_j$ occasions, ${{\varvec{C}}}_j=(C_{1j},\ldots ,C_{n_jj})'$, ${{\varvec{W}}}_j=(Z_j,X_{1j},\ldots ,X_{n_jj})'$, and ${{\varvec{Y}}}_j=(Y_{1j},\ldots ,Y_{n_jj})'$. Then C-MAR becomesAgain, this is covariate-dependent missingness in the sense of Little (1995). Selection cannot depend on $\zeta _j$ because this latent variable is always missing, and we are not conditioning on it.

If selection depends on $\zeta _j$, we can adopt a fixed-effects approach. We now treat $\zeta _j$ as fixed by using indicator (or dummy) variables $I_{rj}$ for units *j* (with $I_{rj}=1$ if $r=j$ and $I_{rj}=0$ otherwise) and omitting the intercept $\alpha$

$$\begin{aligned} Y_{ij} \ = \ \beta X_{ij} + \sum _{r=1}^N \zeta _r I_{rj} + \epsilon _{ij}. \end{aligned}$$

The coefficient $\gamma$ of the time-invariant covariate $Z_j$ cannot be estimated because $Z_j$ is perfectly collinear with the dummy variables for the units. Selection based on $\zeta _j$ now becomes selection based on covariates $I_{rj}$, and $\beta$ can be estimated consistently (see also Verbeek & Nijman, 1992). The requirement for valid inference now becomes4Another advantage of the fixed-effects approach is that it controls for all possible known and unknown time-invariant confounders (e.g., Skrondal & Rabe-Hesketh, 2022).

Adopting a fixed-effects estimator for $\beta$ while not obtaining any inferences for $\gamma$ and $\psi$ can also be viewed as an example of Strategy 3, protective estimation, discussed in Sect. 5. There we describe the standard fixed-effects estimator for random-intercept logistic regression, which is the conditional maximum likelihood estimator. That estimator is valid under C-MAR*. Furthermore, modifying the model and/or discarding more data produces protective estimators under several MNAR mechanisms.

Interestingly, when selection $S_j$ of *units* *j* (instead of unit-occasion combinations) depends on $\zeta _j$ and not on the covariates ${{\varvec{W}}}_j$, i.e., when , the maximum likelihood estimator for the random-intercept model in (3) is consistent for the regression coefficients. The reason is that selection alters only the latent variable distribution $P(\zeta _j|S_j=1)\ne P(\zeta _j)$ and not the conditional response distribution $P({{\varvec{Y}}}_j|{{\varvec{W}}}_j,\zeta _j,S_j=1)=P({{\varvec{Y}}}_j|{{\varvec{W}}}_j,\zeta _j)$ and consistency of the regression coefficients does not rely on correct specification of the random-effects distribution in linear mixed models (Verbeke & Lesaffre, 1997). When the model is modified to a common factor model, by replacing $\zeta _j$ by $\lambda _i\zeta _j$ (with $\lambda _1=1$), replacing $\alpha$ by $\alpha _i$, and removing the covariates, the maximum likelihood estimator of the factor loadings $\lambda _i$ is also consistent when . This result is closely related to factorial invariance (e.g., Meredith, 1964). As pointed out by Skrondal & Rabe-Hesketh (2004, p. 56), consistency requires that anchoring (setting a factor loading to 1) is used for identification instead of factor standardization (setting the variance of the factor to 1) because the variance of the latent variable is different in the selected sample. Choosing anchoring to obtain consistent estimates of the factor loadings can therefore also be seen as a form of protective estimation.

## Strategy 2: Discard More Data

In this section, we return to the scenario with three variables *X*, *Z*, and *Y*, where *Z* is always observed, whereas *X* and *Y* are not always observed, and we are interested in a model for *P*(*X*, *Z*, *Y*). Even if we are interested only in the parameters governing *P*(*Y*|*Z*, *X*), we may want to model the joint distribution by IL methods, making use of AA data, because it is more efficient than CC regression (e.g., Little & Schluchter, 1985). Now R-MAR is required for valid frequentist inference. However, we consider two missingness processes that violate R-MAR and show that we can still obtain valid frequentist inference by discarding more data to *make* the process A-MAR before proceeding with IL inference.

### MNAR-X: X Affects Selection of Y

**Fig. 4:**
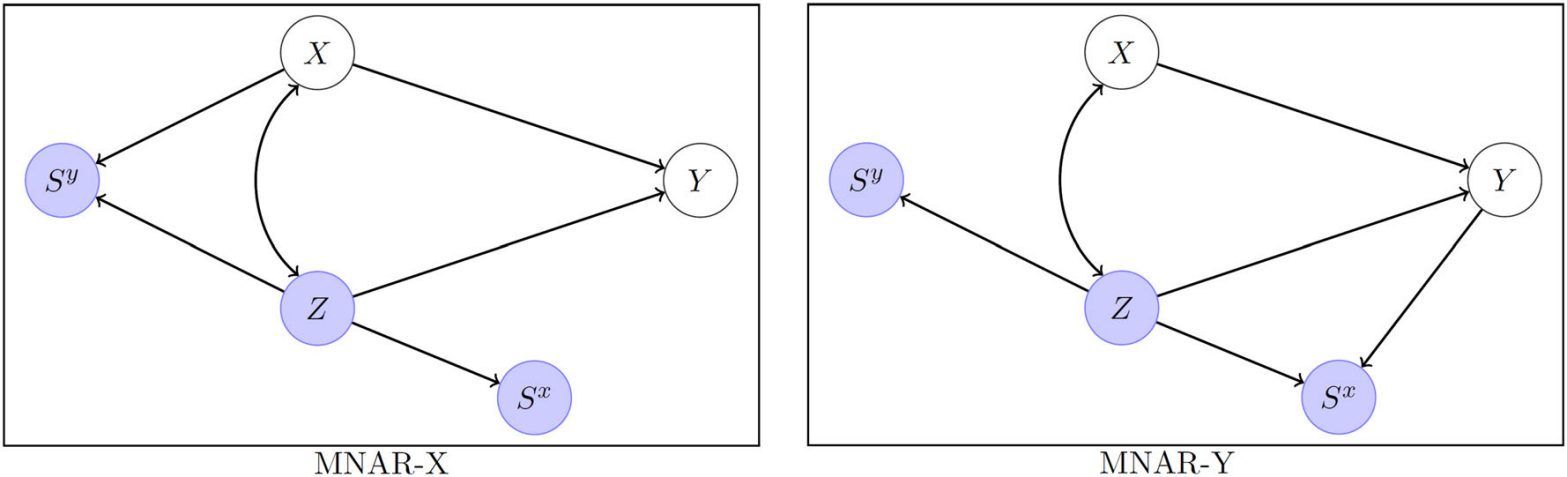
MNAR-X and MNAR-Y mechanisms.

Consider the *m*-graph in the left panel of Fig. 4 with proxy variables not shown. Here, the DAG for *X*, *Z*, and *Y* is compatible with the SEM in Fig. 1, but could correspond to many other statistical models because DAGs are nonparametric. This graph is not strictly a DAG because there is a double-headed arrow between *X* and *Z*, but this arrow could be replaced by a latent variable node with paths to both *X* and *Z*. R-MAR is violated because of the path $X\rightarrow S^y$. However, C-MAR is satisfied because , so we could perform CC regression. However, if we would like to estimate the joint distribution *P*(*X*, *Z*, *Y*), IL methods will not be valid.

#### M-MAR

It turns out that IL methods become valid if we discard *Y* when $S^x=0$, with corresponding modified missingness indictor

$$\begin{aligned} {\dot{S}}^y=\left\{ \begin{array}{cc}0&{}\hbox {if}\ S^x=0\\ S^y&{}\hbox {if}\ S^x=1 \end{array}\right. .\end{aligned}$$

This does not mean deleting *units* when *X* is missing, but just making *Y* missing for the units with missing *X* (but retaining *Z* for these units). We now show that the process for ${\dot{S}}^y$ satisfies A-MAR by factorizing the joint probability of the selection indicators as

$$\begin{aligned} P(S^x\!\!,{\dot{S}}^y|{{\textbf {U}}})&=P(S^x|{{\textbf {U}}})P({\dot{S}}^y|S^x,{{\textbf {U}}}), \end{aligned}$$

where the first term is $P(S^x|{{\textbf {U}}})=P(S^x|Z)$ and the second term is

$$\begin{aligned} P({\dot{S}}^y\!=\!1|S^x,{{\textbf {U}}}) =\ \left\{ \begin{array}{ll}0&{}\hbox {if\ }S^x=0\\ P({\dot{S}}^y\!=\!1|X^{\text {obs}},Z)&{}\hbox {if\ }S^x=1\end{array}\right. . \end{aligned}$$

We see that $P({\dot{S}}^y|S^x,{{\textbf {U}}})=P({\dot{S}}^y|S^x\!\!,X^{\text {obs}},Z)$, so the following condition is satisfied:

5 $$\begin{aligned} \hbox {M-MAR:}\ P(S^x\!\!,{\dot{S}}^y|{{\textbf {U}}}) = P(S^x|Z)P({\dot{S}}^y|S^x\!\!,X^{\text {obs}},Z) = P(S^x\!\!,{\dot{S}}^y|{{\textbf {U}}}^{\text {obs}}).\end{aligned}$$

The idea is that we allow selection of *Y* to depend on *X* if *X* is selected/observed, but when *X* is missing, we make selection of *Y* impossible so that it no longer depends on the unobserved *X*. The M-MAR (*make* MAR) condition is satisfied because we made it so by data deletion. We can think of the selection process as a natural process, represented in the left panel of Fig. 4, followed by deletion of *Y* when *X* is missing by the data analyst. It does not matter for inference that part of the process is man-made. If we imagine that data analysts will behave this way in repeated samples, we have A-MAR and frequentist IL inference is therefore valid.

#### Ordered Factorization

Figure 1(d) in Mohan et al. (2013) corresponds to the *m*-graph in the left panel of Fig. 4 with *Z* removed. Applying their approach (in their Example 3) to our situation, the joint distribution can be factorized as follows:

6 $$\begin{aligned} P(X, Z, Y) = P(Z) P(X|Z) P(Y|X, Z). \end{aligned}$$

Then the terms are estimated sequentially as follows:

Step 1: Estimate *P*(*Z*) by using all units because *Z* is never missing.
Step 2: Estimate *P*(*X*|*Z*) by using only those units with $S^x=1$ (i.e., deleting units with $S^x=0$). This is valid because , so that $P(X|Z)=P(X^*|Z, S^x=1)$.
Step 3: Estimate *P*(*Y*|*X*, *Z*) by using only units with $S^xS^y=1$, i.e., pruning the dataset further by deleting units with $S^y=0$. This is valid because  so that $P(Y|X, Z)=P(Y^*|X, Z, S^x=1 , S^y=1)$.

This last step corresponds to CC regression and is justified because C-MAR is satisfied. Mohan et al. (2013) point out that the *deletion order* matters. Units with missing *X* are deleted in Step 2, followed by deletion of further units, with missing *Y*, in Step 3.

#### M-MAR Versus Ordered Factorization for MNAR-X

We can consider the contribution of a unit to the AA-data log-likelihood after discarding *Y* when *X* missing. Replacing $s^y$ in (2) by ${\dot{s}}^y$, the third term disappears, $(1-s^x){\dot{s}}^y\text {ln}P(z,y)=0$, because $(1-s^x)$ is nonzero only when $s^x=0$, and in this case ${\dot{s}}^y=0$. We therefore have

$$\begin{aligned} L^{\text {joint}}\ =\ {}&s^x{\dot{s}}^y\text {ln}P(x,z,y) + s^x(1-{\dot{s}}^y)\text {ln}P(x,z) + (1-s^x)(1-{\dot{s}}^y)\text {ln}P(z). \end{aligned}$$

Using the factorization in (6), we can rewrite this log-likelihood contribution as

$$\begin{aligned} L^{\text {joint}}\ {}&{}={}\ s^x{\dot{s}}^y[\text {ln}P(y|x,z) + \text {ln}P(x|z) + \text {ln}P(z)] + s^x(1-{\dot{s}}^y)[\text {ln}P(x|z) + \text {ln}P(z)] \\&\quad + (1-s^x)(1-{\dot{s}}^y)\text {ln}P(z) \\&{}={} \text {ln}P(z) + {s^x \text {ln}P(x|z)} + s^x{\dot{s}}^yP(y|x,z). \end{aligned}$$

We can see that information about *P*(*Z*) comes from all units, information about *P*(*X*|*Z*) comes only from the subset of units with $s^x=1$, and information about *P*(*Y*|*X*, *Z*) comes only from the subset of units with both $s^x=1$ and $s^y=1$, exactly as in the sequential estimation proposed by Mohan et al. (2013). Factorization such as shown in (6) also facilitates AA-data maximum likelihood estimation (e.g., Anderson, 1957; Marini et al., 1980), and for this reason (not for achieving consistency) it has been suggested to discard data (Marini et al., 1980, p. 333).

It is instructive to consider why it is necessary to discard values of *Y* when *X* is missing or why including the third term from (2), namely $(1-s^x){s}^y\text {ln}P(z,y)$, in the log-likelihood would lead to inconsistent estimation. Units with $s^x=0$ and $s^y=1$ contribute to this term, but $P(Z,Y|S^x=0,S^y=1)\ne P(Z,Y)$ because $S^y$ is a collider in the graph, so conditioning on it creates a new backdoor path between *Z* and *Y* through *X* and therefore corrupts the joint distribution.

The M-MAR approach is preferable to sequential estimation whenever the goal is to estimate parameters of a parametric model. After deleting *Y* when *X* is missing, estimation can be performed straightforwardly using standard software for IL methods, such as AA-data maximum likelihood estimation, and standard error estimates are produced as a byproduct. In contrast, Mohan et al. (2018) use *m*-graphs to derive sequential estimators for parameters of linear SEMs. Their estimators of regression coefficients are sums of products of estimators of variances and other path coefficients and require complex algorithms to evaluate sequentially. Estimation of standard errors requires further work, such as a delta method or resampling approaches.

### MNAR-Y

For MNAR-Y, shown in the right panel of Fig. 4, the problem is that selection of *X* depends on *Y*, but *Y* is not always observed. It is clear that CC regression cannot be used to estimate *P*(*Y*|*X*, *Z*) because , violating C-MAR.

#### M-MAR

The M-MAR solution here is to delete *X* when *Y* is missing, with corresponding modified missingness indicator,

$$\begin{aligned} {\dot{S}}^x=\left\{ \begin{array}{cc}0&{}\hbox {if}\ S^y=0\\ S^x&{}\hbox {if}\ S^y=1 \end{array}\right. .\end{aligned}$$

We can factorize the joint probability of the selection indicators as

$$\begin{aligned} P(S^x\!\!,{\dot{S}}^y|{{\textbf {U}}})&=P(S^y|{{\textbf {U}}})P({\dot{S}}^x|S^y,{{\textbf {U}}}), \end{aligned}$$

where $$P(S^y|{{\textbf {U}}})=P(S^y|Z)$$, and

$$\begin{aligned} P({\dot{S}}^x\!=\!1|S^y,{{\textbf {U}}}) =\ \left\{ \begin{array}{ll}0&{}\hbox {if\ }S^y=0\\ P({\dot{S}}^x\!=\!1|Y^{\text {obs}},Z)&{}\hbox {if\ }S^y=1\end{array}\right. , \end{aligned}$$

so that

7 $$\begin{aligned} \hbox {M-MAR:}\ P({\dot{S}}^x\!\!,S^y|{{\textbf {U}}}) = P(S^y|Z)P({\dot{S}}^x|S^y\!\!,X^{\text {obs}},Z) = P({\dot{S}}^x\!\!,S^y|{{\textbf {U}}}^{\text {obs}}).\end{aligned}$$

The term omitted from the AA-data log-likelihood is ${\dot{s}}^x(1-s^y)\text {ln}P(x,z)$ because ${\dot{s}}^x=0$ whenever $s^y=0$. This term is problematic because conditioning on $S^x$ produces an additional path between *X* and *Z* through *Y*.

#### Ordered Factorization

For MNAR-Y, the ordered factorization approach by Mohan et al. (2013) is based on the factorization *P*(*Z*)*P*(*Y*|*Z*)*P*(*X*|*Z*, *Y*). Unfortunately, the conditional distribution of interest *P*(*Y*|*X*, *Z*) does not appear directly but can derived from the joint distribution by dividing it by *P*(*X*, *Z*) if $P(X, Z)> 0$. Note that we cannot obtain *P*(*X*, *Z*) directly because $P(X, Z) \ne P(X^*, Z|S^x=1)$, but we can obtain *P*(*X*, *Z*) by marginalizing the joint distribution. In practice, the marginalization will not be straightforward and the resulting distribution may not be a closed-form function of the model parameters of interest. In contrast, M-MAR remains as easy to implement as for MNAR-X and will directly yield estimates of the parameters of interest if the joint distribution is parameterized in terms of *P*(*Y*|*X*, *Z*) as in Fig. 1. Therefore, M-MAR becomes the method of choice for MNAR-Y.

For a model with three variables, we have considered two different R-MAR violations and shown how we can make the missingness A-MAR. With more variables, a general approach would be to identify, for each variable *V*, which other variables have direct paths to $S^v$. If any of these variables are missing for a unit *i*, discard $V_i$. This approach presupposes substantive understanding of the missingness mechanisms and may lead to a considerable loss of data. An alternative approach would be to check whether it is possible to sort the variables so that the missingness pattern is approximately monotone, in the sense that earlier variables are rarely missing for a unit if later variables are not missing for the unit. The next step would be to assess whether it is justifiable to assume that selection of each variable is independent of subsequent variables given the previous variables and their selection indicators. If this does not appear reasonable for a given variable, the variable should be placed later in the sequence as needed. The final step would be to make the missingness monotone. If there are covariates that affect their own selection, we can condition on those variables in the IL method, as described in Sect. 3.2.

### Making Longitudinal Data Monotone

**Fig. 5:**
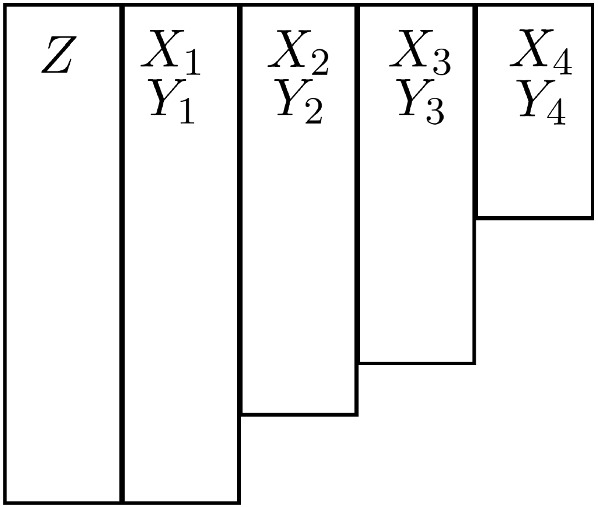
Monotone missingness pattern for longitudinal data.

Returning to a longitudinal setting with the notation of Sect. 3.3, we consider the scenario where the CC indicator $C_{ij}$ for unit *j* at occasion *i* being a complete “case” with $X_{ij}$ and $Y_{ij}$ (as well as $Z_j$) observed depends on the unit’s outcomes at previous occasions. Then A-MAR is violated because those previous outcomes may be missing, unless the missingness patterns are (always) monotone as shown in Fig. 5. Here rows represent units *j* which have been sorted in terms of the occasion when missing data first occur, and the rectangles for ($X_{ij}$, $Y_{ij}$, $i=1, 2, 3, 4$) enclose all units with complete data at occasions 1, 2, 3, and 4.

When missingness is not monotone, we propose *making* the missingness monotone. This means deleting $Y_{ij}$ if any previous $Y_{ij}$ is missing:

$$\begin{aligned} {\dot{C}}_{ij}= \left\{ \begin{array}{cc} 0 &{} \hbox {if} \ \prod _{r=1}^{i-1}C_{rj}=0 \\ C_{ij} &{} \hbox {otherwise} \end{array}\right. . \end{aligned}$$

As in previous subsections, we are exploiting the fact that A-MAR allows for dependencies among selection indicators, and that we can manufacture part of the selection process ourselves. For example, consider the case where selection depends on the previous outcome. Then the new selection mechanism becomes

$$\begin{aligned}&\ P({\dot{C}}_{ij}=1|{{\varvec{Y}}}_j,Z_j,\zeta _j,C_{1j},\ldots , C_{i-1,j})\ = \left\{ \begin{array}{cc}0&{}\hbox {if}\ C_{i-1,j}=0\\ P(C_{ij}=1|Y_{i-1,j}^{\text {obs}},Z_j)&{}\hbox {if}\ C_{i-1,j}=1 \end{array}\right. \end{aligned}$$

and satisfies A-MAR.

Interestingly, the fact that A-MAR allows missingness to depend on other responses for the same unit is often mentioned in the longitudinal data literature, but the point that this requires monotone missingness is rarely mentioned, an exception being Schafer and Graham (2002). In longitudinal data, it is possible that only monotone patterns can occur because having a missing value at an occasion means that the unit has dropped out and cannot re-enter the study. When there are no such barriers to re-entering the study, it is difficult to think of a natural selection mechanism where the previous response causes missingness only when it is observed. Therefore, the A-MAR property cannot be assumed to hold even if the realized missingness pattern is monotone, unless the data analyst imagines that she would make the data monotone in repeated samples.

As mentioned at the end of Sect. 3.3, regression coefficients (or factor loadings) can be estimated consistently in linear mixed models (or factor models) if selection $S_j$ of *units* depends on the random effects (or latent variables) as long as . This result does not hold when there is *item* non-response, where $C_{ij}$ can be 1 for some items (or occasions) *i* and 0 for other items for the same unit *j*. In this latter situation, we can convert item non-response to unit non-response by dropping units with $\prod _i C_{ij}=0$, so that ${\dot{S}}_j=\prod _i C_{ij}$. Then consistency is achieved under the assumption that .

## Strategy 3: Be Protective of (Subsets of) Parameters

### Logistic Regression

Strategy 3 is best introduced for logistic regression, for simplicity with a single covariate $X_i$,

$$\begin{aligned} P(Y_i=1|X_i) = \frac{\exp ( \alpha + \beta X_i)}{1+\exp ( \alpha + \beta X_i)}.\end{aligned}$$

In a case–control study, controls (with $Y_i=0$) are undersampled relative to cases (with $Y_i=1$), also known as outcome-based or retrospective sampling, and selection into the CC sample $C_i=S_i^xS_i^y$ therefore depends on $Y_i$:

$$\begin{aligned} P(C_i=1|X_i,Y_i=1)=P(C_i=1|Y_i=1)&\equiv \pi (1)\\ P(C_i=1|X_i,Y_i=0)=P(C_i=1|Y_i=0)&\equiv \pi (0). \end{aligned}$$

The model for the CC sample becomes

$$\begin{aligned} P(Y_i=1|X_i,C_i=1)&=\ \frac{\pi (1)P(Y_i=1|X_i)}{\pi (0)P(Y_i=0|X_i)+\pi (1)P(Y_i=1|X_i)}\\&=\frac{\pi (1)\exp ( \alpha + \beta X_i)}{\pi (0)+\pi (1)\exp ( \alpha + \beta X_i)}\\&=\frac{\{\pi (1)/\pi (0)\}\exp ( \alpha + \beta X_i)}{1+\{\pi (1)/\pi (0)\}\exp ( \alpha + \beta X_i)}. \end{aligned}$$

It follows that

$$\begin{aligned}\text {logit}[P(Y_i=1|X_i,S_i^x=1,S_i^y=1)]\ =\ \underbrace{[\alpha +\text {ln}\{\pi (1)/\pi (0)\}]}_{\alpha ^*} + \beta X_i,\end{aligned}$$

so the log odds ratio, $\beta$, is estimated consistently by maximum likelihood, whereas the estimator of the intercept $\alpha$ converges to $\alpha ^*=\alpha +\text {ln}\{\pi (1)/\pi (0)\}$. The intercept can be estimated consistently only if $\pi (1)/\pi (0)$ is either known (e.g., by design) or can be consistently estimated, in which case $\text {ln}\{{\widehat{\pi }}(1)/{\widehat{\pi }}(0)\}$ can be included in the logistic regression model as an offset. This result is well-known for case–control designs (e.g., Breslow, 1996).

### Fixed-Effects Logistic Regression for Longitudinal Data

As in Sect. 3.3, we consider a random-intercept model for clustered or longitudinal data, but now with a logit link for a binary outcome variable:

8 $$\begin{aligned} \text {logit}[P(Y_{ij}=1|X_{ij}, Z_j, \zeta _j)]\ =\ \alpha + \beta X_{ij} + \gamma Z_{j} + \zeta _j,\quad \zeta _j\sim N(0,\psi ). \end{aligned}$$

Again, we could replace $\zeta _j$ by a fixed effect to be able to relax the C-MAR requirement to C-MAR* defined in (4), where $\zeta _j$ can directly affect selection. Because of an incidental parameter problem, the fixed-effects estimator is not obtained by including indicator variables for the units as in Sect. 3.3, but by conditional maximum likelihood estimation.

The contribution from unit *j* to the conditional likelihood, given the sum of the outcomes for the unit, $\tau _j=\sum _i Y_{ij}$, is

9 $$\begin{aligned} P({{\varvec{Y}}}_j | \sum Y_{ij}\!=\!\tau _j, {{\varvec{W}}}_{j}, \zeta _j) \ = \ \frac{ \prod _{i=1}^{n_j} \exp ( \beta X_{ij} )^{Y_{ij}} }{ \sum _{{{\varvec{d}}}_j \in {{{\mathcal {B}}}}_j} \prod _{i=1}^{n_j} \exp ( \beta X_{ij} )^{d_{ij}} }, \end{aligned}$$

where ${{{\mathcal {B}}}}_j \! = \! \{ {{\varvec{d}}}_j \! = \! (d_{1j},\ldots ,d_{nj})^{\prime }$ | $d_{ij} = 0$ or 1, and $\sum _{i} d_{ij}\!=\!\tau _j \}$, or in words, ${{{\mathcal {B}}}}_j$ is the set of all vectors of length $n_j$ with binary elements that sum to $\tau _j$. This set can be obtained by permuting the elements of ${{\varvec{Y}}}_j$. Note that the between-unit component of the model, $\alpha + \gamma Z_j + \zeta _j$, cancels out due to conditioning on the sufficient statistic $\tau _j$.

When there are missing data, we let ${{{\mathcal {I}}}}_j$ be the set of occasions for unit *j* when outcomes are observed and redefine ${{{\mathcal {B}}}}_j$ as

$$\begin{aligned}{{{\mathcal {B}}}}_j \! = \! \left\{ {{\varvec{d}}}_j | d_{ij} = 0\ \hbox {or}\ 1, i \in {{{\mathcal {I}}}}_j,\ \hbox {and}\ \sum _{i \in {{{\mathcal {I}}}}_j} d_{ij} = \tau _j \right\} .\end{aligned}$$

The conditional likelihood contribution from unit *j*, conditioning on the vector of selection indicators ${{\varvec{C}}}_{j}$, is:10If selection does not depend on observed outcomes, given missing outcomes and random intercepts, $P({{\varvec{C}}}_j|{{\varvec{Y}}}_j^{\text {obs}}, {{\varvec{Y}}}_j^{\text {mis}}, {{\varvec{W}}}_{j}, \zeta _j)=P({{\varvec{C}}}_j|{{\varvec{Y}}}_j^{\text {obs}}, {{\varvec{W}}}_{j}, \zeta _j)$, then the integrals in the numerator and denominator are identical and we obtain the standard conditional likelihood in (9).

If selection depends on the current outcome only,the integrals in the numerator and denominator of (10) becomeandrespectively. Taking the first product in square brackets out of each integral, the ratio of these integrals becomes the ratio of the products in square brackets, givingThen we can be protective of $\beta$ by including occasion-specific intercepts $\alpha _i$ in the original model in (8) that represent $\alpha +\text {ln}(\pi _{i}(1)/\pi _{i}(0))$,

$$\begin{aligned} \text {logit}[P(Y_{ij}=1|X_{ij}, Z_j,\zeta _j)]\ =\ \alpha _i +\ \beta X_{ij} + \gamma Z_{j} + \zeta _j. \end{aligned}$$

Skrondal and Rabe-Hesketh (2014) show that if selection $C_{ij}$ at occasion *i* depends on the outcome $Y_{i-1,j}$ at the previous occasion, a consistent estimator for $\beta$ is obtained by either analyzing complete units (across time, $\prod _i C_{ij}=1$) only and including occasion-specific intercepts $\alpha _i$, or by allowing the occasion-specific intercepts to take on different values for different missingness patterns across time (through interactions between indicators for occasions and indicators for the missingness patterns). If selection depends on both the previous and current outcomes, a consistent estimator for $\beta$ is obtained by analyzing complete units (across time) with sum of outcomes equal to $\tau _j=1$ or $\tau _j=n-1$ and allowing the occasion-specific intercepts to take different values for $\tau _j=1$ and $\tau _j=n-1$.

## Concluding Remarks

One message of this address is that complicated procedures, such as multiple imputation instead of CC regression, are often not necessary and could perform worse than simple approaches. Even when MAR assumptions are violated, joint modeling of the missingness and substantive processes can often be avoided. By making conditional independence assumptions regarding the missingness process, we can instead derive simple estimators that do not require explicit modeling of the missingness process.

We also made the point that confusion persists in the literature regarding MAR and MCAR and what assumptions are needed for different estimators. We are therefore excited that Mohan, Pearl and co-authors are developing a completely new framework for investigating missing data problems that we believe holds strong promise, especially if it is adopted in statistics. Mohan and Pearl (2021) provide an accessible overview of their approach for a statistical audience.

Our main new contribution is to propose discarding more data as one way to handle MNAR problems. We justified this approach by purely relying on the A-MAR assumption and recognizing that it is immaterial whether the entire selection mechanism is due to nature or whether part of it is man-made. We also showed the connection of our estimator to Mohan et al.’s sequential estimator based on their ordered factorization theorem. It seems that this connection is not at all obvious given the much more cumbersome estimators developed for SEMs in Mohan et al. (2018).

We made conditional independence assumptions regarding the missingness process which may sometimes be justifiable based on an understanding of the phenomena being studied. However, some of these conditional independencies are testable. Ji et al. (2023) propose such tests, show that they are powerful, and that test-based estimators (chosen based on the results of the conditional independence tests) have smaller mean squared error than the naive AA-data maximum likelihood estimator for SEMs in a wide range of conditions.

## Footnotes

1 The missingness process $P({{\textbf {s}}}_i|{{\textbf {u}}}_i)$ depends on parameters $\varvec{\phi }$ and MAR requires that the equality holds for all possible values of $\varvec{\phi }$. We added the *i* subscript which is permitted because we will assume that ${{\textbf {S}}}_i$ and ${\varvec{U}}_i$ are i.i.d.
